# *ZmNRAMP4* Enhances the Tolerance to Aluminum Stress in *Arabidopsis thaliana*

**DOI:** 10.3390/ijms23158162

**Published:** 2022-07-25

**Authors:** Hongjie Li, Ning Wang, Wanpeng Hu, Weina Yan, Xinwu Jin, Yan Yu, Chengfeng Du, Chan Liu, Wenzhu He, Suzhi Zhang

**Affiliations:** 1 Key Laboratory of Biology and Genetic Improvement of Maize in Southwest China of Agricultural Department, Ministry of Agriculture, Maize Research Institute, Sichuan Agricultural University, Chengdu 611130, China; lemontrain@163.com (H.L.); wnyaoyaoxinghe@163.com (N.W.); huwanpeng814642@163.com (W.H.); ywn18395409252@163.com (W.Y.); jinxinwu121419@163.com (X.J.); yuyanbigfish@163.com (Y.Y.); duyeye1936551448@163.com (C.D.); liuchan0910@163.com (C.L.); 2 Crop Research Institute, Sichuan Academy of Agricultural Sciences, Chengdu 610066, China; wenzu_he@163.com

**Keywords:** Al toxicity, *ZmNRAMP4*, plasma membrane, maize, metal transporter

## Abstract

Aluminum (Al) toxicity causes severe reduction in crop yields in acidic soil. The natural resistance-associated macrophage proteins (NRAMPs) play an important role in the transport of mineral elements in plants. Recently, *OsNrat1* and *SbNrat1* were reported specifically to transport trivalent Al ions. In this study, we functionally characterized *ZmNRAMP4*, a gene previously identified from RNA-Seq data from Al-treated maize roots, in response to Al exposure in maize. *ZmNRAMP4* was predominantly expressed in root tips and was specifically induced by Al stress. Yeast cells expressing *ZmNRAMP4* were hypersensitive to Al, which was associated with Al accumulation in yeast. Furthermore, overexpression of *ZmNRAMP4* in *Arabidopsis* conferred transgenic plants with a significant increase in Al tolerance. However, expression of *ZmNRAMP4*, either in yeast or in *Arabidopsis*, had no effect on the response to cadmium stress. Taken together, these results underlined an internal tolerance mechanism involving *ZmNRAMP4* to enhance Al tolerance via cytoplasmic sequestration of Al in maize.

## 1. Introduction

Aluminum (Al) is the most abundant metal element in the Earth’s crust [1]. About 50% of the Earth’s arable land is acidic. In acidic soil (pH < 5.0), Al exists in the form of trivalent Al (Al^3+^), which is toxic to plants, especially at high concentrations [2]. Therefore, Al toxicity is an important limiting factor for crop yield in acidic soil [3].

To cope with the high availability of soil Al, plants have evolved two mechanisms involving external exclusion and internal tolerance [4,5]. External exclusion of Al via exudation of organic acid anions from roots is an important Al resistance mechanism in many plants. External exclusion of Al via exudation of organic acid anions from the roots is an important Al tolerance mechanism in many plant species. Malate and citrate transporters have been functionally characterized in many species, including *AtMATE* and *AtMLT1* in *Arabidopsis*, *OsFRDL2* and *OsFRDL4* in rice, *SbMATE* in sorghum, and *TaALMT1* in wheat [6]. With regard to an internal detoxification mechanism, some transporters associated with Al tolerance can be so classified. For example, STAR1 and STAR2 form a transporter protein complex that is responsible specifically for efflux of UDP-glucose, which is likely to be used to modify the cell wall [7]. In *Arabidopsis*, *AtSTAR1* and *ALS3* are involved in Al tolerance [8,9]. Overexpression of *OsPIN2*, which encodes an auxin transporter, can decrease the binding of Al to the cell wall [10]. Certain transcription factors (*AtSTOP1* [11], *OsART1* [12], *VuNAR1* [13], *WRKY47* [14], *HvHOX9* [15], *GsMAS1* [16] and abscisic acid (ABA)-stress and ripening (*ASR5*) [17]) and ROS-related genes (*ZmAT6*) [18] are also involved in plant Al stress response.

NRAMP proteins comprise a group of highly conserved integral membrane proteins widespread among bacteria, animals, and plants [19]. The genomes of sorghum, rice, maize, and *Arabidopsis* contain 7, 7, 8, and 6 NRAMP members, respectively [20]. NRAMP proteins play a major role in the transport of mineral elements in plants, including the metal ions Fe^2+^, Mn^2+^, Cd^2+^, Zn^2+^, Co^2+^, and Cu^2+^ [21]. For example, *OsNRAMP1* transports Cd, As, and Fe, but not Mn [22]; *OsNRAMP5* is the main transporter for Cd^2+^ influx in rice [23,24]; *AtNRAMP3* and *AtNRAMP4* transport Fe^2+^ and Mn^2+^ in *Arabidopsis* [25]; *BcNRAMP1* is a plasma membrane-localized protein and transports Mn and Cd [26]; and *FeNRAMP5* is a transporter for the uptake of Mn and Cd [27]. Interestingly, two NRAMP members, *OsNrat1* and *SbNrat1*, transport Al^3+^ specifically but not divalent metal ions, such as Fe^2+^, Mn^2+^, and Cd^2+^, and are required for a prior step of final Al detoxification through vacuolar sequestration of Al [3,28,29]. The aim of the present study was to functionally characterize the role of *ZmNRAMP4*, which was previously identified from RNA-Seq data for Al-stressed maize roots, in Al transport in maize. The results showed that *ZmNRAMP4* was upregulated by Al treatment. Combined heterologous expression in yeast and *Arabidopsis* indicated that *ZmNRAMP4* is an Al transporter and its expression is stimulated by Al. Overexpression of *ZmNRAMP4* enhanced the Al tolerance of transgenic *Arabidopsis* plants via Al influx in the roots.

## 2. Results

### 2.1. Cloning and Phylogenetic Analysis of ZmNRAMP4

**Figure 1:**
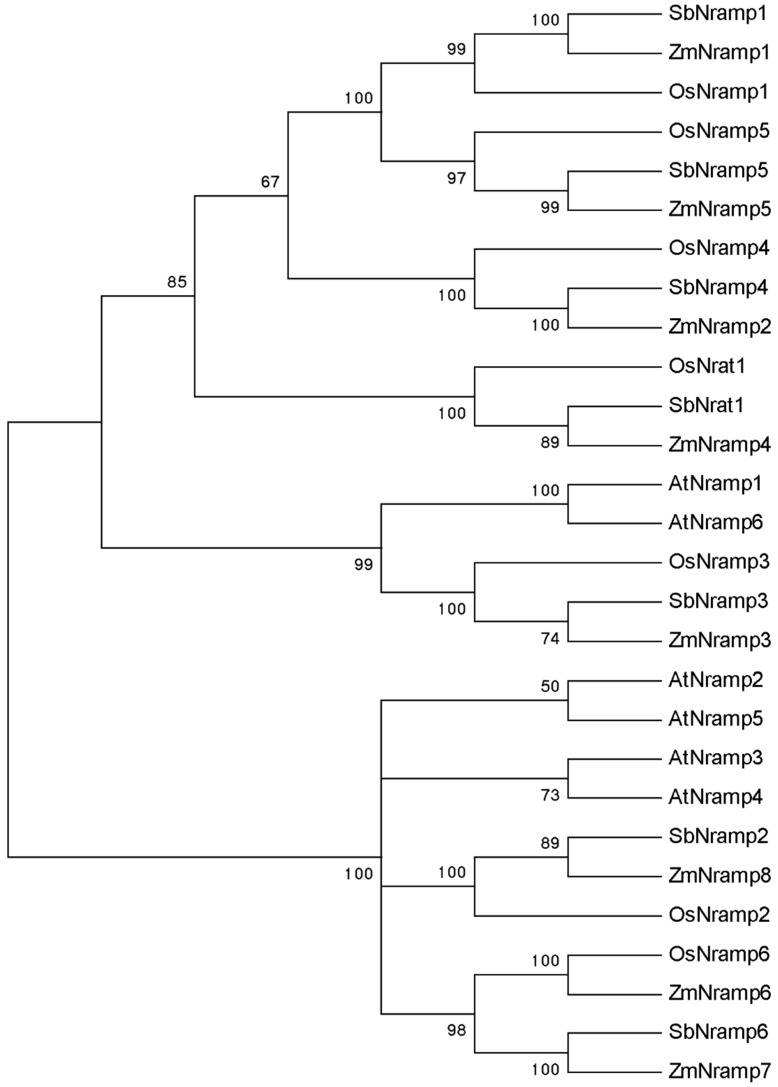
Phylogenic tree of *ZmNRAMP4* with NRAMPs from *Arabidopsis*, rice, sorghum, and maize. The information of accession number and species for NRAMPs is as follows, including AtNRAMP1 to AtNRAMP6 (AT1G80830, AT1G47240, AT2G23150, AT5G67330, AT4G18790, AT1G15960), OsNRAMP1 to OsNRAMP6 (Os07g15460, Os03g11010, Os06g46310, Os07g15370, Os12g39180, Os01g0503400), OsNrat1 (Os02g0131800), SbNRAMP1 to SbNRAMP6 (XP_002459640, XP_002465667, XP_002438846, XP_021317241, XP_002461772, XP_002464246), SbNrat1 (XP_002451480), ZmNRAMP1 to ZmNRAMP8 (Zm00001d005479, Zm00001d007397, Zm00001d014391, Zm00001d015133, Zm00001d019327, Zm00001d030846, Zm00001d033367, Zm00001d048129).

*ZmNRAMP4* was cloned from the Al-tolerant maize inbred line 178 (Figure S1). The gene encoded a putative protein of 556 amino acids, which belongs to the NRAMP family of maize. The multiple sequence alignment showed that *ZmNRAMP4* included all conserved elements of NRAMP members, such as Motif A and Motif B, and had the highest identity with *SbNrat1* of 96% (Figure S2). A phylogenetic tree for NRAMP members was composed of five main clades. Notably, *ZmNRAMP4* was placed in the same clade as *OsNrat1* and *SbNrat1*, two previously reported transporters of trivalent Al ions, but was separated from divalent ion transporters (Figure 1).

### 2.2. Expression Patterns of ZmNRAMP4

**Figure 2:**
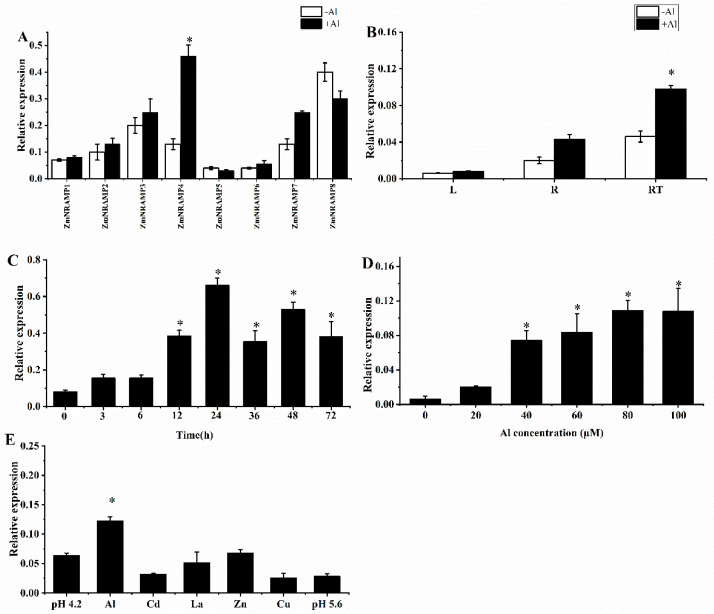
Expression pattern of the *ZmNRAMP4* gene. (**A**), Relative expression of *ZmNramp* gene family in the presence of Al. (**B**), Relative expression of *ZmNRAMP4* in root tips (0–1 cm), root regions (behind removed root tips, 1–10 cm), and leaves. (**C**), Time-dependent expression of *ZmNRAMP4* in the root tips. The seedlings were cultured in the nutrient solution added with 60 µM AlCl_3_ for 0, 3, 6, 12, 24, 36, 48, and 72 h. (**D**), Expression analysis of *ZmNRAMP4* under different concentration of Al. Seedlings were treated for 6 h by 0, 10 μM, 20 μM, 40 μM, 60 μM, 80 μM, and 100 μM Al. (**E**), Expression of *ZmNRAMP4* in response to other metals. Seedlings were exposed to a solution containing 60 µM AlCl_3_, 10 µM LaCl_3_, 100 µM ZnSO_4_, 2 µM CuCl_2_, and 30 µM CdCl_2_ or pH 4.2 for 12 h. Asterisk indicated significant difference from control at *p* < 0.05 by Tukey’s test with three independently biological replicates.

To explore if the eight ZmNRAMP members were stimulated by Al stress, the expression patterns of all eight *ZmNRAMP* genes were investigated. The results revealed that *ZmNRAMP4* was the only *ZmNRAMP* gene that was induced by Al stress (Figure 2A). Therefore, the expression pattern of *ZmNRAMP4* in response to Al stress was studied further. The Al-induced expression of *ZmNRAMP4* was predominantly detected in the root and was highest in the root tip (Figure 2B). A time-course experiment showed that *ZmNRAMP4* expression increased after Al exposure and peaked at 24 h, and thereafter decreased with prolonged treatment (Figure 2C). The expression of *ZmNRAMP4* was significantly affected by the AlCl_3_ concentration; a broad range of AlCl_3_ concentrations (40–100 µM) stimulated *ZmNRAMP4* expression effectively and 80 μM AlCl_3_ induced the optimum stimulation (Figure 2D). In addition, *ZmNRAMP4* expression was specifically affected by Al rather than other metal ions, such as Zn, Cu, La, or Cd, as well as low pH (Figure 2E).

### 2.3. Subcellular Localization of ZmNRAMP4

**Figure 3:**
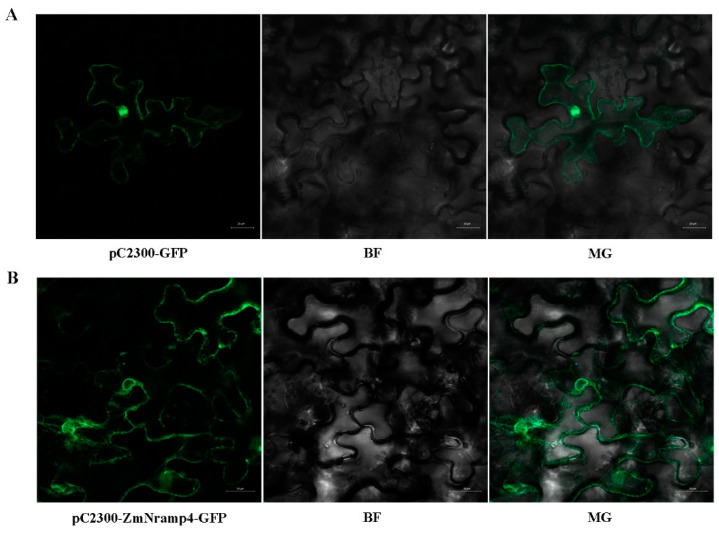
Subcellular localization of *ZmNRAMP4* in tobacco leaves. The *ZmNRAMP4*-GFP fusion protein was transiently expressed in the leaves of *N. benthamiana* (Scale bar: 50 μm).

To visualize the subcellular localization of *ZmNRAMP4*, ZmNRAMP4-GFP was transiently expressed in *Nicotiana benthamiana* leaves. The green fluorescent signal of GFP of the control vector was ubiquitous throughout the cell (Figure 3A). In contrast, the ZmNRAMP4-GFP signal was only detected at the plasma membrane (Figure 3B), consistent with the prediction of *ZmNRAMP4* as a putative metal ion transporter.

### 2.4. Functional Expression of ZmNRAMP4 in Yeast

**Figure 4:**
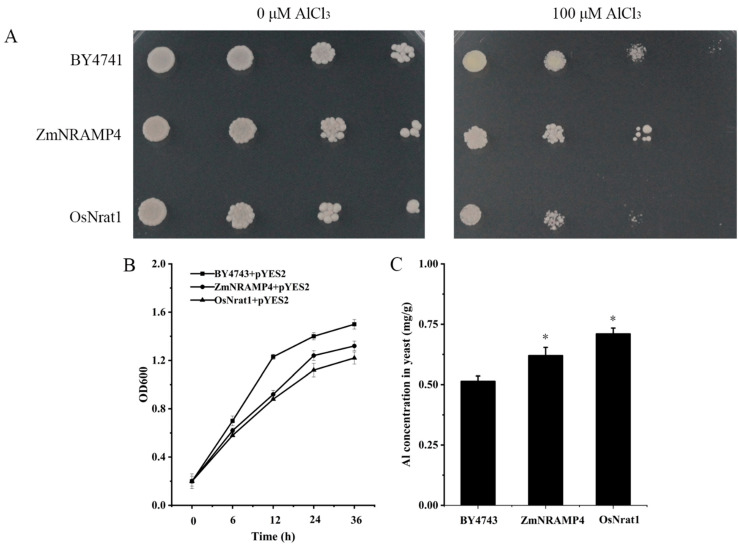
Al transport activity of *ZmNRAMP4* in yeast. (**A**), the growth of yeast cells carrying *ZmNRAMP4* and *OsNrat1* on LPM medium plate with 0 or 100 μM AlCl_3_. (**B**), Growth curves of yeast cells transformed with empty vector pYES2, pYES2-*OsNrat1*, or pYES2-*ZmNRAMP4* in a galactose containing LPM liquid medium with or without 60 μM AlCl_3_. (**C**), Al concentration in yeast. Asterisk indicated significant difference from control at *p* < 0.05 by Tukey’s test with three independently biological replicates.

To investigate the Al transport ability of ZmNRAMP4, *ZmNRAMP4* was expressed in the yeast strain BY4741. *OsNrat1*, an Al transporter in rice, its expression vector, and the empty vector were used as a positive control and negative control, respectively. In the absence of Al, all yeast cells, either carrying *ZmNRAMP4*, *OsNrat1*, or the empty vector, showed similar growth rates (Figure 4A). However, compared with the negative control, the growth of yeast cells expressing *ZmNRAMP4* or *OsNrat1* were slightly inhibited in the presence of Al (Figure 4A), suggesting that Al influx into the yeast cells occurred. Moreover, the yeast cells expressing *ZmNRAMP4* or *OsNrat1* accumulated a higher concentration of Al (Figure 4C), accompanied by stronger inhibition of growth (Figure 4B) when compared with the negative control. We also investigated the Cd transport activity of ZmNRAMP4 in yeast because some NRAMP proteins are involved in the transport of other metal ions. The pYES2-*ZmNRAMP4* construct or the empty vector pYES2 were expressed both in a Cd-sensitive mutant strain Δycf1 and the wild-type strain BY4741. No growth difference was observed among the transformed yeasts in the absence of Cd stress (Figure S3). In response to treatment with 50 µM CdCl_2_, BY4741 yeast cells carrying *ZmNRAMP4* grew similarly to those carrying the empty vector. In contrast, Cd severely inhibited the growth of Δycf1 yeast cells transformed with the empty vector but not cells transformed with pYES2-*ZmNRAMP4* (Figure S3). These results revealed that *ZmNRAMP4* has transport activity for Al but not Cd in yeast.

### 2.5. Overexpression of ZmNRAMP4 Enhanced Al Tolerance of Arabidopsis Plants

**Figure 5:**
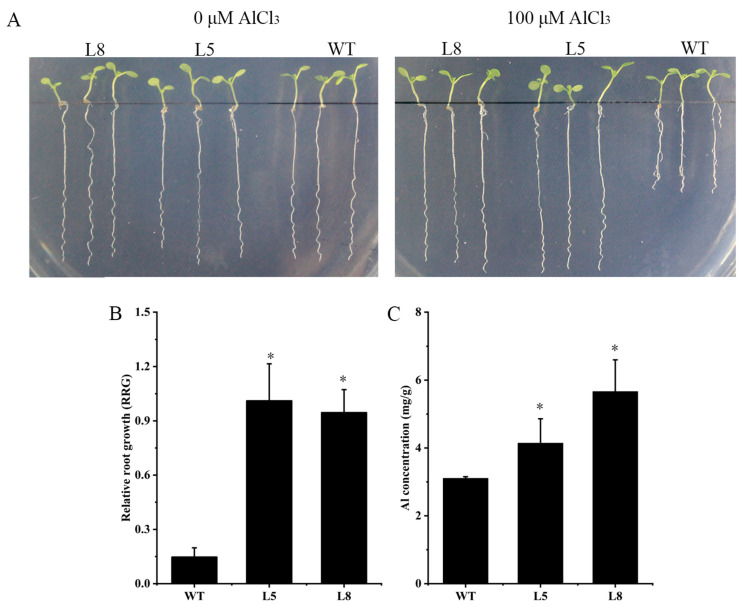
Phenotype of ZmNRAMP4-overexpressed transgenic *Arabidopsis* plants under Al stress. (**A**) Al sensitivity, (**B**) relative root growth (RRG), and (**C**) Al concentration in wild-type and ZmNRAMP4 transgenic plants. The 14-day-old seedlings were exposed to one-fifth Hoagland solution (pH 4.7) containing 100 μM Al for 12 h. Asterisk indicated a significant difference from control at *p* < 0.05 by Tukey’s test with three independently biological replicates.

**Figure 6:**
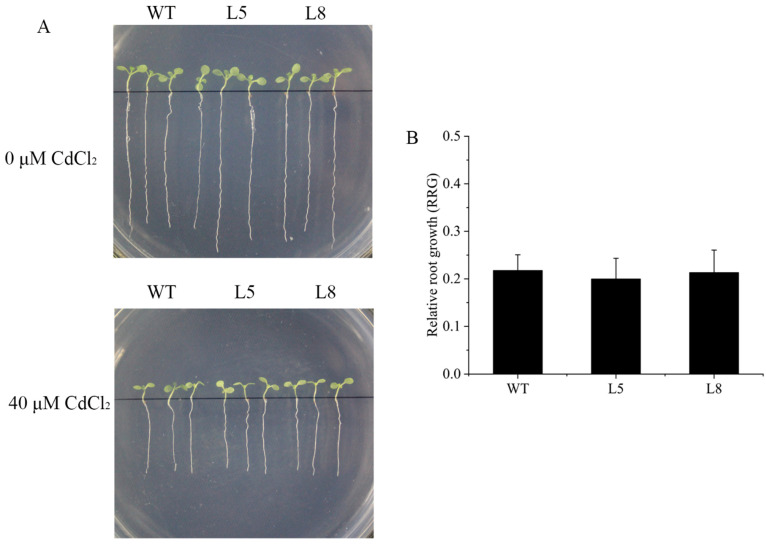
Phenotype of ZmNRAMP4-overexpressed transgenic *Arabidopsis* plants under Cd stress. (**A**) Cd sensitivity and (**B**) relative root growth (RRG) in wild-type and ZmNRAMP4 transgenic plants. Relative root growth (RRG) of wild-type and *ZmNRAMP4* transgenic plants under 0 or 50 µM CdCl_2_.

To determine whether *ZmNRAMP4* affected Al tolerance in other plants, *ZmNRAMP4* was overexpressed in *Arabidopsis* under the control of the *35S* promoter. The Al-tolerant phenotype conferred by *ZmNRAMP4* was investigated in two transgenic lines that highly expressed *ZmNRAMP4* (Figure S4). Under normal condition, root growth of transgenic plants was comparable with that of the wild type, whereas the roots were longer than those of the wild type after Al exposure (Figure 5A). Similarly, the relative root growth of the transgenic lines was significantly higher than that of the wild type under Al stress (Figure 5B). In addition, the transgenic lines accumulated greater concentrations of Al in the root than the wild type (Figure 5C). However, overexpression of *ZmNRAMP4* did not affect the root growth of the transgenic *Arabidopsis* plants under Cd stress (Figure 6). These results demonstrated that overexpression of *ZmNRAMP4* in *Arabidopsis* conferred a notable increase in Al tolerance, but had no effect on tolerance of Cd stress.

## 3. Discussion

Most NRAMP proteins are capable of transporting Cd and essential metals, such as Mn and Fe [30]. Recent studies have shown that *OsNrat1* and *SbNrat1* have transport activity for Al [3]. ZmNRAMP4 is a candidate gene for Al tolerance QTL co-localization and upregulated after 6 h of Al exposure in the root tip [31]. However, the functional characterization of *ZmNRAMP* genes in response to Al stress remains unclear. To verify the function of *ZmNRAMP4*, we investigated the effects of *ZmNRAMP4* expression on metal tolerance and accumulation in yeast and *Arabidopsis*.

In rice, *OsNrat1* expression is stimulated rapidly by Al, particularly in the root, and expression is maintained at a low level in the shoot. Furthermore, the stimulation by Al is specific because other metals do not induce *OsNrat1* expression. The expression pattern of *ZmNRAMP4* was consistent with that of *OsNrat1* but differed notably from that of *SbNrat1*, which was not responsive to Al regardless of the Al concentration and exposure duration [3]. Given that *ZmNRAMP4* was placed in the same subclade as *OsNrat1* and *SbNrat1* in the present phylogenic analysis (Figure 1), the difference in expression is probably attributable to differences among cereal species in the fine-tuning of environmental adaptation during evolution. This suggestion is consistent with the evolution of diverse functions among *NRAMP* members in the transport of metal ions, such as Fe^2+^, Mn^2+^, Cd^2+^, Zn^2+^, Co^2+^, and Cu^2+^.

Different from other Nramp members that could transport divalent metals, *OsNRAT1* specifically transports trivalent aluminum [32]. In the present study, NRAMP transporters were resolved into five clades in a phylogenetic analysis, of which *ZmNRAMP4* was placed in the same clade as the previously reported Al^3+^ transporters *OsNrat1* and *SbNrat1* (Figure 1). The metal binding site of NRAMP proteins is characterized by two conserved sequence motifs, i.e., the Asp–Pro–Ser–Asn motif (motif A) and the Ala–Ile–Ile–Thr motif (motif B) [33]. The Nrat1-type motif B is both effective and essential for Al transport by OsNrat1, as one crucial determinant of Al selectivity [34]. Missense mutations of amino acids in motif B of Al-tolerant rice lines reduce Al uptake in yeast cells, and the full function of *OsNrat1* in Al influx is dependent on the most closely matched sequence of motif B [32]. Therefore, the exactly matched sequence of motif B in ZmNRAMP4 may be the predominant determinant of Al selectivity (Figure S2). In the present study, *ZmNRAMP4*-expressing yeast cells exhibited transport activity for Al but not Cd, which was consistent with accumulation of a higher concentration of Al. This was also the case for the Al influx transporters OsNrat1 and SbNrat1 when expressed in yeast [3]. In addition, the plasma membrane localization of ZmNRAMP4 was compatible with its function as a metal ion transporter.

*NRAMP* genes have been reported to be widely involved in the transport of metal ions. Recent studies have shown that overexpression of *SbNrat1* recovers the Al tolerance of the rice mutant *osnrat1* [3]. Similarly, overexpression of *OsNRAT1* improves the Al tolerance of transgenic *Arabidopsis* and results in a significant increase in Al content [32]. In the present study, overexpression of *ZmNRAMP4* in *Arabidopsis* conferred significantly elevated Al tolerance compared with the control, but had no effect on Cd tolerance. Consistent with the Al influx transport function of ZmNRAMP4, the roots of transgenic *Arabidopsis* plants accumulated higher concentrations of Al. The expression of endogenous *AtALS1* is elevated (Figure S5), consistent with significantly enhanced expression of *NRAT1* and Al accumulation in transgenic *Arabidopsis* roots [32]. Given that AtALS1 has the capacity to sequester the cytosolic Al in the vacuole, it is speculated that orthologs of AtALS1 coordinate with ZmNRAMP4 to enhance the Al tolerance of maize via Al sequestration.

## 4. Materials and Methods

### 4.1. Plant Materials and Growth Conditions

The Al-tolerant maize inbred line 178 was used in this experiment [18]. Seeds were sterilized in 10% NaClO for 15 min, washed three times with deionized water, then cultured in an incubator at 28 °C. After germination, the seedlings were transplanted to pots filled with Hoagland’s nutrient solution and grown in a greenhouse maintained at 28/20 °C under a 16 h/8 h (day/night) photoperiod. The nutrient solution was replaced every 3 days.

### 4.2. Phylogenetic Analysis

The sequences of NRAMP family members were retrieved from the ARAMEMNON plant membrane protein database (http://aramemnon.botanik.uni-koeln.de/index.ep; accessed on 12 December 2018). A phylogenetic tree was constructed using the neighbor-joining algorithm with MEGA 5.0.

### 4.3. Relative Expression Analysis of ZmNRAMP4

For metal stress treatment, seedlings at the three-leaf stage were treated individually with 60 µM AlCl_3_, 10 µM LaCl_3_, 100 µM ZnSO_4_, 2 µM CuCl_2_, 30 µM CdCl_2_, or pH 4.2 for 12 h. To examine the expression pattern of *ZmNRAMP4*, maize seedlings were exposed to Al (0, 10, 20, 40, 60, 80, or 100 µM) for 12 h or 60 µM AlCl_3_ for different durations (0, 3, 6, 12, 24, 36, 48, and 72 h). Root and leaf samples were individually collected, rapidly frozen in liquid nitrogen, and stored at −80 °C for RNA extraction. The primers used for RT-PCR of *ZmNRAMP4* are listed in Supplementary Table S2.

### 4.4. Subcellular Localization of ZmNRAMP4

The cDNA of *ZmNRAMP4* was subcloned into the expression vector p*C2300-GFP* under the control of the *35S* promoter using the primers listed in Supplementary Table S2. The p*C2300-GFP* construct was introduced into *Agrobacterium tumefaciens* strain EHA105. *Agrobacterium*-mediated transient expression assays were conducted in *Nicotiana benthamiana* leaves [35].

### 4.5. Mental Ion Transport Activity of ZmNRAMP4 in Yeast

The coding sequence of *ZmNRAMP4* was cloned into the pYES2 vector (negative control) to generate the construct p*YES2*-*ZmNRAMP4*. The construct p*YES2*-*OsNrat1* was generated to serve as a positive control. The yeast strains used in this study were BY4741 (*MATa his2Δ0 met15Δ0 ura3Δ0*) and the Cd-sensitive strain Δycf1 (*MATa his2Δ0 met15Δ0 ura3Δ0 YOL122c::KanMX4*). Aluminum and Cd sensitivity was tested on agar, whereas Al uptake was measured in liquid culture. For Al-sensitivity evaluation, the constructs p*YES2*-*ZmNRAMP4* and p*YES2*-*OsNrat1* and the empty vector pYES2 were transformed into yeast strain BY4741 and cultured on solid medium (LPM with galactose as the carbon source) supplemented with 0 or 100 µM Al buffered with 5 mM succinic acid. For Cd-sensitivity evaluation, p*YES2*-*ZmNRAMP4* and pYES2 were transformed into yeast strains BY4741 and Δycf1, and cultured in liquid SD-U medium supplemented with 0 or 50 µM Cd. For estimation of Al uptake in liquid culture, transformants were selected on uracil-deficient medium and grown in synthetic complete yeast solution using glucose as the carbon source. Cells at the mid-exponential phase were harvested, transferred to LPM medium using galactose as the carbon source, and were cultured for 2 h. Next, AlCl_3_ was added to the medium at the final concentration of 60 µM Al. After incubation for 4 h with shaking, the cells were harvested by centrifugation, washed three times with deionized water, and then digested with 2 M HCl. The concentration of Al in the digest solution was determined by inductively coupled plasma–mass spectrometry.

### 4.6. Overexpression of ZmNRAMP4 in Arabidopsis thaliana

*ZmNRAMP4* was inserted into the pC2300 vector under the control of the *35S* promoter. Transgenic plants were screened with kanamycin (40 mg/L) and confirmed by PCR analysis. Two dependent T_3_ transgenic lines with elevated expression levels of *ZmNRAMP4* were used for assessment of Al tolerance (Figure S4).

### 4.7. Evaluation of Al Tolerance

To quantify the effect of Al toxicity, the wild type and two transgenic lines were surface sterilized and germinated for 4 days on solid Murashige and Skoog (MS) medium. Seedlings of uniform growth were transferred to half-strength MS solid medium supplemented with AlCl_3_ (0 or 100 μM) or CdCl_2_ (0 or 25 μM) and cultured for 7 days. The root length of the seedlings was measured individually with a ruler before and after treatment.

### 4.8. Statistical Analysis and Reproducibility

All treatments were repeated at least three times. Statistical analysis including student’s *t* test was performed using the SPSS software. The figures were drawn using the Origin 8.0 software (OriginLab Corporation, Northampton, MA, USA).

## 5. Conclusions

We investigated a ZmNRAMP4 gene encoding a plasma membrane-localized Al influx transporter from the Al-tolerant maize inbred line 178. Its expression is significantly upregulated in response to Al exposure, but not by other metals, in maize roots. ZmNRAMP4 confers Al influx activity in yeast cells. Ectopic overexpression of *ZmNRAMP4* in *Arabidopsis* enhances the Al tolerance of transgenic plants. The results suggested that *ZmNRAMP4* may increase resistance to Al toxicity via cytoplasmic sequestration of Al in transgenic *Arabidopsis*.

## Data Availability

All data generated or analyzed during this study are available within the article or upon request from the corresponding author.

## Supplementary Materials

The following supporting information can be downloaded at: https://www.mdpi.com/article/10.3390/ijms23158162/s1.
