# Supplementary material for: ZmNRAMP4 Enhances the Tolerance to Aluminum Stress in Arabidopsis thaliana
Source: Int J Mol Sci. 2022 Jul 25;23(15):8162. doi: 10.3390/ijms23158162 (PMC9331102; doi:10.3390/ijms23158162)
Supplement: Supplementary file 1 [file ijms-23-08162-s001.zip › ijms-1809614-supplementary/Supplemental/Table S1.pdf]

Table S1 The basic information of *ZmNRAMP4*

Gene name	Locus tag	ORF length (bp)
ZmNRAMP4	*Zm00001d015133*	1816
