# Supplementary material for: ZmNRAMP4 Enhances the Tolerance to Aluminum Stress in Arabidopsis thaliana
Source: Int J Mol Sci. 2022 Jul 25;23(15):8162. doi: 10.3390/ijms23158162 (PMC9331102; doi:10.3390/ijms23158162)
Supplement: Supplementary file 1 [file ijms-23-08162-s001.zip › ijms-1809614-supplementary/Supplemental/Table S2.pdf]

Table S2 The primers used in this study

Program	Forward	Reverse
ZmNramp4 for gene cloning	5'-TCCCCCGGGCAGGCAGGCACATCACTAGC-3'	5'-GCTCTAGAGAATGAGCCAGCACGCCTCT-3'
ZmNramp4 for subcellular localization	5'-TCCCCCGGGCAGGCAGGCACATCACTAGC-3'	5'-GCTCTAGACGTGGAAGCATCAGCCAGGT-3'
ZmNramp4 for RT-PCR	5'-TGGTGGCGTTCCTCATCAAC-3'	5'-CGTACACGACGGAGCTCGAC -3'
ZmGAPDH for RT-PCR	5'-CTGGTTTCTACCGAGTTCCTTG-3'	5'-CGGCATACACAAGCAGCAAC-3'
OsNrat1 for gene cloning	5'-GGGGTACCATGGAAGGGACTGGTGAGAT-3'	5'-GCTCTAGACTACATGGAAGCATCGGCAA-3'
AtNramp4 for gene cloning	5'-GGGGTACCTGACAAATCTATCCCTGCGC-3'	5'-GCTCTAGAACTCACTCATCATCCCTCTG-3'
