# Supplementary figures and images for: ZmNRAMP4 Enhances the Tolerance to Aluminum Stress in Arabidopsis thaliana
Source: Int J Mol Sci. 2022 Jul 25;23(15):8162. doi: 10.3390/ijms23158162 (PMC9331102; doi:10.3390/ijms23158162)

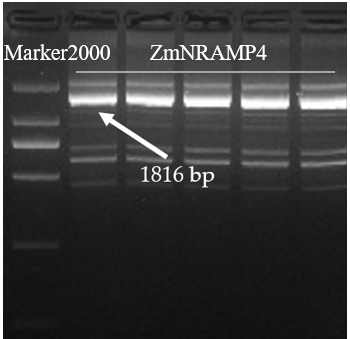

Supplement: Supplementary file 1 [file ijms-23-08162-s001.zip › ijms-1809614-supplementary/Supplemental/Fig. S1.png]

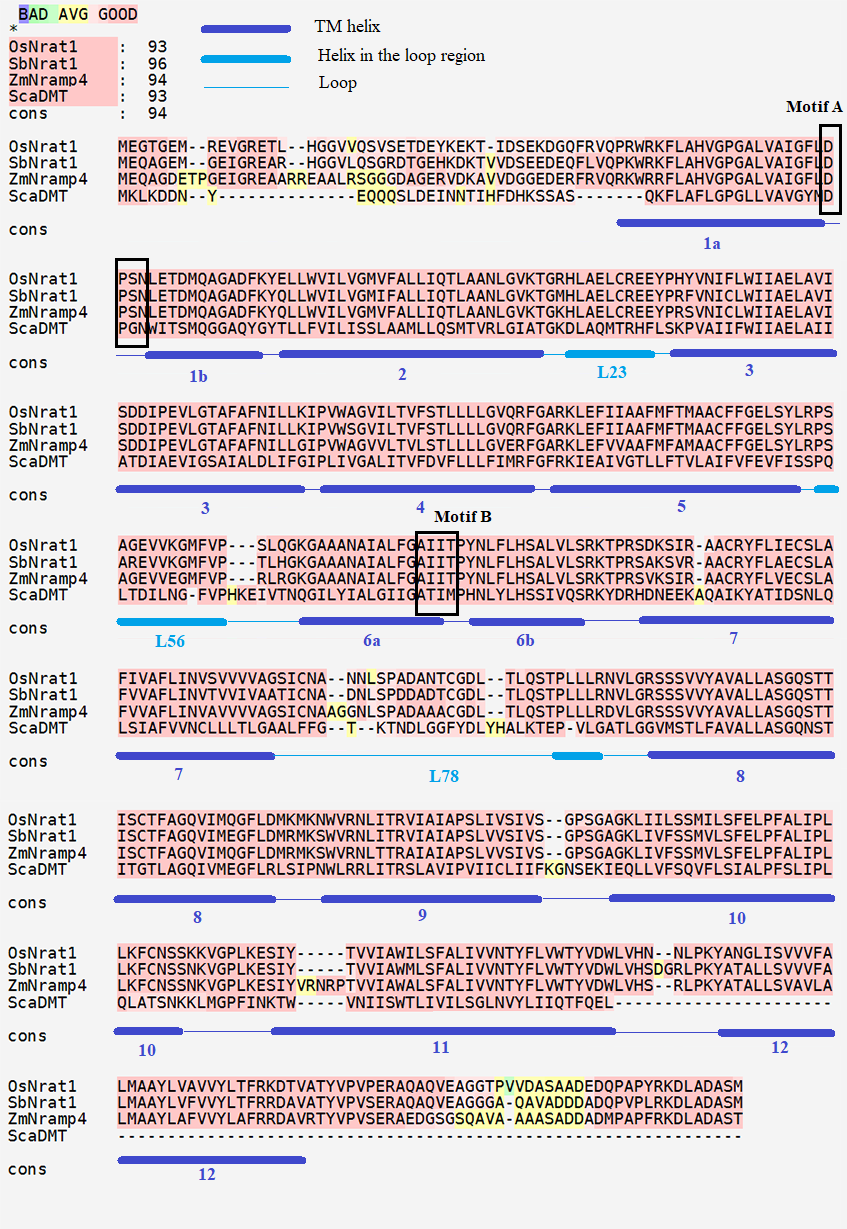

Supplement: Supplementary file 1 [file ijms-23-08162-s001.zip › ijms-1809614-supplementary/Supplemental/Fig. S2.tif]

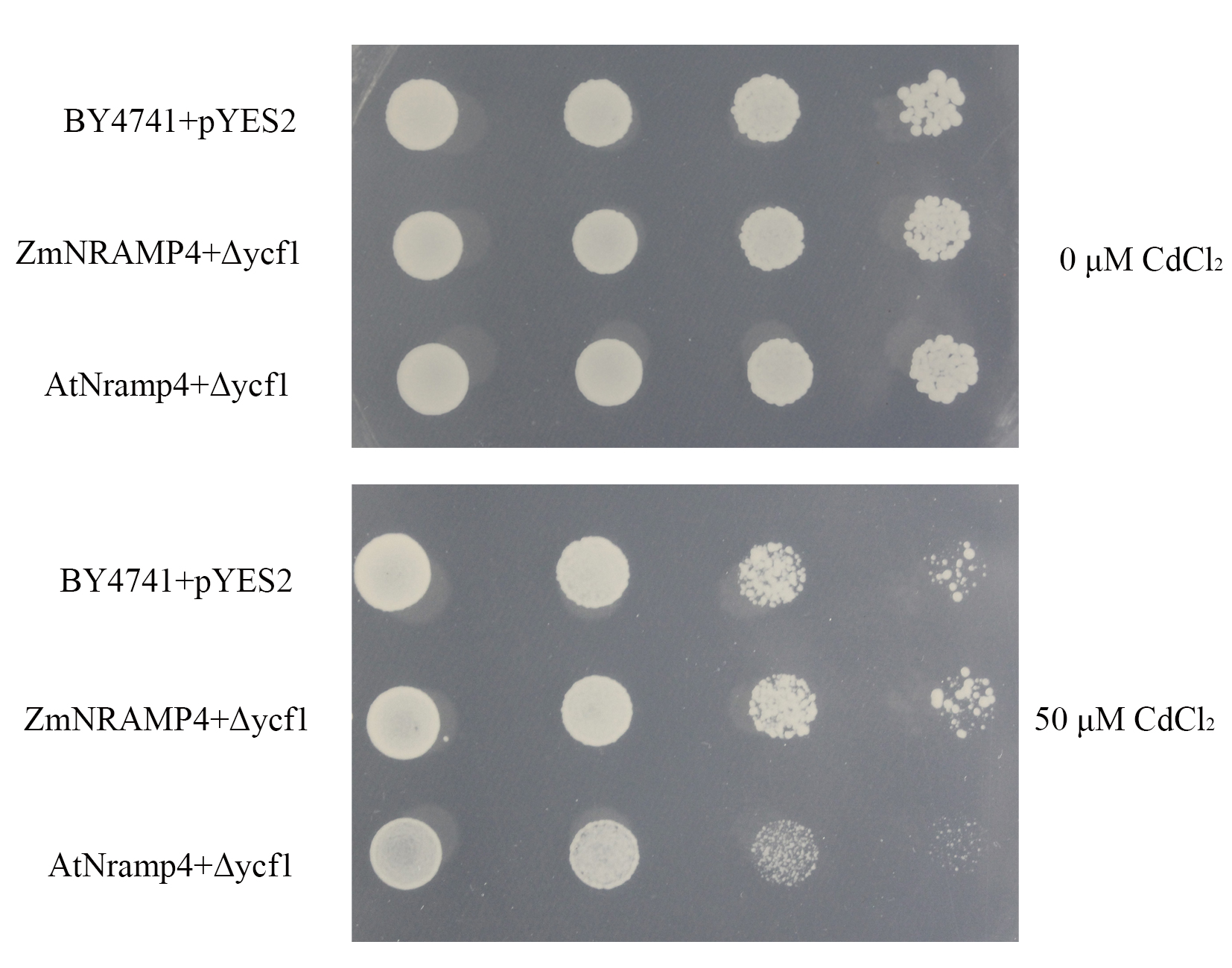

Supplement: Supplementary file 1 [file ijms-23-08162-s001.zip › ijms-1809614-supplementary/Supplemental/Fig. S3.tif]
